# Using script theory to cultivate illness script formation and clinical reasoning in health professions education

**Published:** 2015-12-11

**Authors:** Stuart Lubarsky, Valérie Dory, Marie-Claude Audétat, Eugène Custers, Bernard Charlin

**Affiliations:** 1 Centre for Medical Education and Department of Neurology, McGill University, Montreal, QC; 2 Centre for Medical Education and Office of Assessment and Evaluation, Undergraduate Medical Education, McGill University, Montreal, QC; 3 Département de médecine familiale et de médecine d’urgence et CPASS (Centre de Pédagogie Appliquée aux Sciences de la Santé), Faculté de médecine, Université de Montréal et Unité des internistes généralistes et pédiatres (UIGP), Faculté de médecine, Université de Genève; 4 University Medical Center at Utrecht, Netherlands Center for Research and Development of Education; 5 Department of Surgery et CPASS (Centre de Pédagogie Appliquée aux Sciences de la Santé), Faculté de médecine, Université de Montréal

## Abstract

Script theory proposes an explanation for how information is stored in and retrieved from the human mind to influence individuals’ interpretation of events in the world. Applied to medicine, script theory focuses on knowledge organization as the foundation of clinical reasoning during patient encounters. According to script theory, medical knowledge is bundled into networks called ‘illness scripts’ that allow physicians to integrate new incoming information with existing knowledge, recognize patterns and irregularities in symptom complexes, identify similarities and differences between disease states, and make predictions about how diseases are likely to unfold. These knowledge networks become updated and refined through experience and learning. The implications of script theory on medical education are profound. Since clinician-teachers cannot simply transfer their customized collections of illness scripts into the minds of learners, they must create opportunities to help learners develop and fine-tune their own sets of scripts. In this essay, we provide a basic sketch of script theory, outline the role that illness scripts play in guiding reasoning during clinical encounters, and propose strategies for aligning teaching practices in the classroom and the clinical setting with the basic principles of script theory.

## Introduction

*Script theory* proposes an explanation for how information is stored in and retrieved from the human mind to influence individuals’ interpretation of events in the world.^1^ Each time it processes a scene, the brain begins by retrieving relevant prior knowledge from memory and using it as a basis for building a model*,* or representation, of the perceived object or event. It then relies on this model to make predictions about what information it should be receiving from the world.^2^ By this account, our brains create meaning by actively comparing the attributes of mental models with the features of an actual scene until one model’s predictions fit well enough to enable us to take appropriate action in the world. The sets of pre-compiled knowledge that give rise to mental models of real-world objects and events are called ‘schemas’ or ‘scripts’.^1^

Since interpretation depends heavily on prior knowledge, it stands to reason that the composition and structure of an individual’s scripts are pivotal for influencing which signals he attends to and how he acts within the world. This assertion is no less true in the health professions, where a clinician’s medical scripts (‘illness scripts’) are thought to be a key determinant of his clinical reasoning acumen and performance in the clinical setting. Studies of expertise development in medicine have consistently shown that those considered to be ‘experts’ are distinguished not by their superior problem-solving skills, nor by their enhanced capacity for memory retrieval, but by the *content* and *organization* of their knowledge base – that is, by the set of individualized scripts they have acquired through learning and experience.^3^,^4^

How clinical and biomedical knowledge gets ‘scripted’ in the brains of learners during medical training is therefore of paramount concern for ensuring that they maintain a steady trajectory toward proficiency in clinical reasoning. In this essay, we provide a basic sketch of script theory, outline the role that ‘illness scripts’ play in guiding reasoning during clinical encounters, and propose strategies for aligning teaching practices in the classroom and the clinical setting with the basic principles of script theory.

### Script theory: what are ‘scripts’?

Imagine a person entering a restaurant and spotting an elephant crouched in the corner. This person is likely to be surprised. Having frequented many restaurants, she has developed certain expectations about the way a restaurant scene should unfold. For example, she expects to perceive food being served and paid for, and individuals waiting on and clearing tables. Her mental representation of a restaurant scene, or *restaurant script*, contains information she holds to be true based on her prior experiences. Her mind is not a blank slate each time she enters a restaurant; rather, her restaurant script instantly springs to mind, generating hypotheses about what signals her senses ought to be receiving, and how she should act in response to them.

Simply put, a *script* is a set of interconnected concepts that allows individuals to make predictions about how a particular event or sequence of events is likely to play out.^1^ Some attributes of a script are more typical of the event in question, or are more likely to actually occur in the real world, than others. Such central or core attributes have a strong influence on a person’s expectations. ‘Waiter’, for example, is an integral attribute of most individuals’ restaurant script; the physical embodiment of the attribute – an actual waiter in a restaurant – is unlikely to bewilder the observer. Since typical or probable script attributes are often seen together, they tend to become associated in memory: when one such attribute (e.g. ‘plate’) is detected in the world, the other (‘cutlery’) is expected to be found nearby. Some attributes, like ‘food service’, are so likely to be found in the real-world instantiation of the event represented by a particular script that their presence may be inferred by default. Peripheral script attributes, on the other hand, may be conceptualized as variables or empty ‘slots’ waiting to be filled in by information from the setting (e.g. ‘salad bar’).^4^

Script theory holds that our brains interpret the world by comparing the attributes of the mental models it creates with the features of an actual scene, checking for consistencies and discrepancies, patterns and irregularities.^1^,^4^ When a perfect or close ‘fit’ exists between the attributes of a mental script and the details of a real-world scene, interpretation occurs quickly, automatically, and effortlessly in the form of instance or pattern recognition.^5^ However, when there is a mismatch between predicted and actual data an individual aptly registers surprise, and initiates a search for an explanation of the anomalous information. This type of search demands slower, more laborious cognitive processing than that required to make sense of routine or typical situations.^5^

Possessed of a good general sense of what to expect in a given setting, an individual can focus her finite attentional resources primarily on the unusual or surprising elements in her environment. Scripts therefore permit humans to decipher the world by making rapid initial judgments about a scene, immediately capturing its gist, and then homing in on the elements that require more careful or deliberate cognitive processing.^1^

In common language, the term ‘script’, in the sense of a ‘screenplay’ (which provides instructions to an actor) or an ‘operational program’ (which provides instructions to a computer), connotes a relatively inflexible entity. Mental scripts, on the other hand, are comparatively dynamic, versatile structures. Rather than an expanding set of loosely-hanging facts, maturing scripts should be conceptualized as flexible, richly organized networks of knowledge that permit rapid interpretation and efficient action.^6^ As an individual gathers new information from the world, her scripts become tailored, pruned, restructured, updated and refined, adapting themselves for use in similar future situations.

### Scripts in medicine: ‘Illness scripts’

Let us consider a physician who is asked to evaluate a patient who is having headaches. When the patient enters his office, he takes note of a young woman who appears to be in some discomfort. She clutches the left side of her head and informs him that the headache ‘started slowly’. These perceptual cues instantly call to mind his *migraine script*: the network of interconnected clinical knowledge he has accumulated through prior experiences learning about and caring for patients with migraines.

Health professionals, like restaurant patrons, rely on mental models to help them make sense of unfolding situations.^7^ *Illness scripts* are specialized knowledge structures that link clinically relevant information about general disease categories, specific examples of diseases, and conditions that enable diseases to flourish in living beings.^8^ The concepts forming the ‘nodes’ of these knowledge networks include pathophysiological mechanisms, contextual factors, and symptoms, and signs of disease. (These have been referred to as *faults*, *enabling conditions*, and *consequences*, respectively 7,16). The idiosyncratic nature of illness scripts, which differ for each clinician according to his or her own prior learning experiences and recollections of patient encounters, accounts for the observation that different medical experts often approach similar clinical problems in variable ways.^9^

### Script activation

According to theory, one or more relevant illness scripts are deployed from a clinician’s mental database in response to early prompts, both verbal (‘started slowly’) and nonverbal (young woman, clutching left side of head, office environment), that he picks up from the patient and the clinical setting.^10^ This process, called *script activation*, generally occurs below the threshold of conscious awareness.^11^ The physician’s activated scripts subconsciously frame his expectations about which signs, symptoms, and background characteristics the patient is likely or not to exhibit, just as his restaurant scripts underlie his expectations about likely restaurant observations and occurrences.

Working from his migraine script, the physician might anticipate finding in this patient a signature pattern of core attributes such as headaches that are ‘severe’, ‘pulsatile’, and associated with ‘light sensitivity’. By virtue of their probability of occurrence together in patients with migraines, these integral, strongly interconnected attributes of his migraine script become automatically co-activated in his mind as a single unit alongside triggered associates (‘young’, ‘female’, ‘gradual onset’, and ‘unilateral’).

### Clinical data interpretation

Thus far, most of the action around scripts has occurred inside the physician’s head, where his intuitions and early perceptions have, in a split second, given rise to a mental representation of a potential migraine scenario. Does his migraine script accurately reflect the reality of the present encounter? Should he take further steps to verify its explanatory power? After all, being wrong could be costly, and at the moment he does not feel pressured to develop an immediate action plan (as emergency physicians, for example, often do). The physician might thus choose to engage in an active search for additional clinical information. *Clinical data interpretation* involves assessing the extent to which new bits of collected data are (or are not) consistent with the attributes of an activated script.^11^

With a few pointed questions, the physician might garner support for his initial diagnostic hypothesis (‘This represents a case of migraine’) if he discovers through direct inquiry a constellation of features (headaches that are ‘severe’, ‘pulsatile’, etc.) that aligns well with his *a priori* expectations about how patients with migraines tend to present. If these expectations are met, he might assume that other associated features of his migraine script (e.g. ‘is relieved by sleep’) are also likely to characterize this patient’s condition, and therefore do not require independent corroboration. He reasons rapidly and effortlessly, luxuriating in mental shortcuts, because there is little ambiguity surrounding the case; nothing he has found violates his preconceived notions about patients with migraines.

However, the unexpected finding of ‘fever’ would automatically trigger the mobilization of an alternate knowledge structure to the physician’s mind: his *brain abscess script*. The clinical data at his disposal will now have to be interpreted in light of at least two competing illness scripts. Faced with this clinical dilemma, he will marshal further information through focused observation, inquiry, and investigation, weighing the impact of the data he collects on the status of his activated scripts.^11^

As new information (narrative history, physical examination findings, or laboratory data, for example) comes to light, additional scripts might become activated, jockeying for a position of dominance in his mind, while others might become attenuated or deactivated.^12^ The physician will continue to gather and weigh information, searching for patterns and irregularities, until he judges that the features of the case match the attributes of one (or more) of his activated scripts closely enough to enable him to develop a working diagnosis and proceed with appropriate investigations, treatment interventions, and counseling. In a process referred to as *script instantiation*, his collection of scripts will become updated to accommodate the specifics of this particular case.^4^ When the next patient enters the room, his previously active scripts are dismissed from working memory, and scripts that are pertinent to the new case immediately flood his mind.

### Summary: Illness scripts and clinical reasoning

In short, script theory proposes that health professionals draw from organized knowledge structures called illness scripts to guide their reasoning during clinical encounters. Script activation refers to the automatic retrieval of one or more relevant scripts from memory in response to early cues from the patient and the clinical setting. Activated scripts are the source of a clinician’s expectations throughout the encounter. Clinical data interpretation involves gauging the ‘fit’ between collected data and activated illness scripts – that is, between actual and expected features of a case. Script instantiation describes the process of filling a script’s empty ‘slots’ with actual information from the context or with information retrieved from memory, and filing it away for future use.

The cognitive processing required to interpret and process data in this fashion varies in rate and level of conscious oversight – from instance or pattern recognition (rapid, nonanalytic) to controlled reasoning (slow, analytic) – depending in part on the quality and scope of a clinician’s existing collection of scripts, and in part on various situational factors such as the complexity, ambiguity, stakes, and time constraints of the clinical problem at hand.^13^,^14^ As a clinician’s repertoire of illness scripts grows, he becomes increasingly likely to possess models that square with the many diverse clinical situations he encounters, enabling him to disambiguate and navigate them swiftly and at relatively minimal cognitive expense.^15^

## Implications for medical education

As we have seen, script theory places *knowledge organization* (rather than generic problem-solving skill) at the foundation of clinical reasoning. Applied to medical education, it holds that learning based on improving the quality of knowledge representations (rather than improving generic clinical reasoning strategies) is fundamental to the development of expertise in diagnosis.^3^ In other words, a learner’s ability to reason through cases will depend on the way that acquired clinical knowledge becomes encoded in his memory. ‘Well-encoded’ medical knowledge is bundled into mental networks (i.e., illness scripts) composed of yoked clinical concepts whose linkages facilitate their rapid mobilization, in aggregate, at the right time and in the right place.

Illness scripts play a key role in supporting key clinical reasoning skills that learners in the health professions must acquire, such as generating differential diagnoses and interpreting clinical data. A conceptually-rich, well-organized illness script is primed to be triggered by a host of relevant cues (‘script activation’), rendering the learner more apt to bring useful prior knowledge to bear on the clinical problem at hand. A learner’s ability to evaluate clinical data and discriminate between competing hypotheses (‘clinical data interpretation’) will improve as the associative links between typical or probable clinical concepts within his illness scripts strengthen, and those between atypical or improbable concepts weaken or dissolve. A broad catalogue of instantiated scripts will develop in learners exposed to a diverse array of patient problems (‘script instantiation’).

A critical goal in medical education, then, is to help learners acquire the necessary building blocks for constructing suitable representations of the numerous and varied situations they are likely (or even unlikely) to encounter, and to ensure that these concepts are strategically linked to facilitate retrieval in the proper clinical contexts. Viewed in this light, script-based education is a concept-forming, link-building enterprise.

Consider the embryogenesis and development of a migraine script in the mind of a medical learner. Before entering medical school, learners may already harbour, through personal experience or exposure to popular media, rudimentary conceptions (and often misconceptions) about certain diseases and their treatments. But for most medical learners entering traditional health professions curricula, substantive knowledge about disease states first begins to accrue in the classroom setting during the pre-clinical years.

### Script-based teaching in the classroom setting

The classroom can and should be viewed as a conducive setting for developing the germinal clinical and biomedical concepts upon which students’ scripts will be elaborated. Three established instructional methods specifically designed to promote early concept formation and linkage – problem-based learning, self-explanations and concept mapping – align particularly well with the basic tenets of script theory.

Problem-based learning (PBL) is based on the principle that active involvement in the elaboration of one’s own knowledge networks leads to deeper understanding and better organization of knowledge for future retrieval.^16^ PBL favors reliance on ‘deep’ learning strategies (e.g. linking new knowledge to prior knowledge) over ‘superficial’ learning strategies (e.g. rote learning and cramming). Learners engaging in PBL should be exposed to authentic and varied clinical problem-solving situations through which they can learn the discriminating features of examples from similar and different disease categories (e.g. migraine vs. brain abscess).^17^ PBL-style learning activities are therefore well-suited for strengthening burgeoning links between concepts, i.e. the core ‘scaffolds’ upon which illness scripts are built.

Self-explanations refer to the internal conversations learners are encouraged to have with themselves as they read texts and solve problems.^18^ Self-explanation has been shown to induce learners to attend to material in a meaningful way, i.e. to link together pieces of information present in the study materials, to integrate new information into existing prior knowledge, and to appropriately restructure knowledge representations.^19^ In their work on self-explanation, Chamberland et al. reported that learners asked to generate self-explanations when reasoning through complex cases demonstrated better diagnostic performance than those who refrained from engaging in internal dialogue, even when no feedback on the self-explanations was provided by a supervisor.^20^

Concept maps are schematic representations of sets of integrated ideas linked by words to create meaning.^21^ Concept mapping could be used as a classroom tool for helping learners link new knowledge with previous knowledge, and hence for fostering meaningful learning. Teachers could, for example, create classroom exercises in which learners are encouraged to create graphical representations of medical concepts and their interrelationships.^22^ By depicting how concepts such as ‘severe’, ‘pulsatile’, and ‘cerebral blood flow’ tend to associate, learners can render knowledge links visually apparent to their teachers by creating concept maps of their own evolving migraine scripts.^23^ Such tools can also be useful for demonstrating how features of a case might overlap several distinct scripts.

### Script-based teaching in the clinical setting

The clinical setting, however, remains the optimal milieu for cultivating the development and refinement of illness scripts.^24^,^25^ Early contact with real patients would serve to ensure that a learner is exposed to – and has an opportunity to develop scripts for – a wide, mixed spectrum of clinical presentations. Evidence from cognitive psychology suggests that human beings are adept at learning from the similarities and differences between even a few cases.^26^ Novice learners should be exposed to patients with common diagnoses (e.g. migraine) and typical presentations to ensure early construction of solid script templates for disease prototypes.^27^ For learners at any stage of development, rarer disease presentations (e.g. brain abscess) should be brought to attention to enable the establishment of scripts where none are likely to exist.

Some learners - often at intermediate levels of training - have accumulated considerable funds of knowledge, but have not yet assimilated this knowledge into well-structured scripts adapted for use in clinical problem-solving situations. Clinician teachers can help learners by attempting to make their own scripts transparent. Think-aloud strategies, for example, can reveal detailed contents of idiosyncratic scripts, and therefore respond to learners’ often unformulated questions about the particular history-taking and examination methods of their clinical teachers.^28^ The features of a clinical presentation that are *not* sought (e.g. ‘is relieved by sleep’) are often as significant as those that are, and learners should be made privy to the process by which their teachers assign ‘default’ status to certain features when reasoning through a case. How teachers weigh features of a case that overlap several scripts should also be clearly articulated for the benefit of learners.

To further promote acquisition and linking of conceptual knowledge in the clinical setting, clinician-teachers might direct learners toward relevant sources of information and encourage them to read about patients’ problems in a manner consistent with the script approach.^19^ When reading about patients’ illnesses in textbooks or journal articles, for example, learners might be stimulated to reflect upon and provide explanations to questions such as: “How important is the finding ‘light sensitivity’ to the diagnosis of migraine?”; “What is the significance of discovering *this* finding (e.g. ‘unilateral’) in the presence of *that* finding (‘fever’)?”; or “If this finding (e.g. ‘gradual onset’) is discovered, how will my thinking about this case change?” During bedside teaching rounds, clinical teachers might adopt specific questioning techniques aimed at strengthening the organization of learners’ knowledge.^29^ Questions probing propositional knowledge (e.g. “Can you name three causes of unilateral headache?”) could instead be framed to reinforce associative learning and script development (e.g. “How might your differential diagnosis be affected by discovering that the headache is unilateral?”). Such link-building strategies can be useful for integrating new knowledge into organized scripts for application to future clinical cases.

Direct observation of performance in the clinical setting is a valuable source of evidence of script development.^30^,^31^ From the questions that learners ask patients, for example, clinician-teachers can gauge whether the learner appears to be following an appropriate thread of thought based on several competing scripts (e.g. migraine script, brain abscess script, meningitis script, etc.). This information can then be used by clinical supervisors to provide specific feedback, reinforcing appropriate script elements and correcting erroneous ones.^32^ The structure and semantic features of case presentations can also provide information about which scripts were activated during a clinical encounter, and how they were used to guide clinical data interpretation.^33^

### Assessment-enhanced teaching and learning

Finally, testing for learning, or “assessment-enhanced teaching and learning,” has been demonstrated in several studies, both in general and medical education, to improve retention for and organization of study material, compared to not being tested or studying alone.^3^,^34^ Widely-used assessment methods based on solving clinical vignettes (e.g. clinical MCQs or extended-matching items) may provide valid evidence of the organization of learners’ knowledge.^35^ More recent instruments have been designed to probe learners’ script activation and data interpretation more directly. The script concordance test, for example, focuses on data interpretation by asking learners to estimate the impact of new information on a suggested hypothesis.^36^,^37^ Clinical reasoning problems seek to measure both script activation and data interpretation using a mix of open-ended and multiple-choice questions similar to the script concordance test.^38^ Direct evidence of knowledge organization can also be provided by assessing learners’ concept maps.^39^

## Discussion

Derived from cognitive psychology, script theory offers a glimpse into the inner workings of a model-building brain as it attempts to interpret signals from the outside world. Transferred to the medical sphere, script theory sheds light on the intricate relationship between clinical reasoning and knowledge organization. Our goal has been to outline several established strategies for aligning teaching practices in the classroom and in the clinical setting with the basic principles of script theory.

Although the list is by no means exhaustive, the common aim of these techniques is to hone the predictive machinery of the brain by fostering in learners the development of organized knowledge networks - illness scripts - that are readily accessible in relevant contexts through multiple memory pathways, and whose influence on reasoning results in appropriate interpretation and efficient action during patient care.

At a deeper level, our desire is to promote the development of *script-conscious* clinician-educators and learners. ‘Script consciousness’ implies an explicit awareness, acquired through guided instruction, of fundamental insights from script theory. We propose that, armed with a working knowledge of the script concept, script-conscious clinician-teachers may be better equipped to guide learners through the pivotal process of illness script formation and refinement and away from the potential pitfalls in clinical reasoning that arise from faulty script development. Although there exists no evidence to support the value of ‘script-consciousness’, evidence from script theory itself provides some indirect justification: just as basic science knowledge is thought to help physicians make sense of clinical practice,^40^ so too might basic knowledge about script theory help educators make sense of educational practice. This is consistent with current conceptions of excellence in clinical teaching: not only do excellent teachers perform their tasks well, but they do so with an understanding of why they are doing what they are doing.^41^

Our treatment of clinical reasoning and knowledge organization in this essay is limited in several regards. For one, our depiction of reasoning in the clinical sphere places disproportionate emphasis on interpretation of purely ‘clinical’ data (symptoms, signs, laboratory data, and the like). We pay scant tribute to the myriad other considerations that influence a clinician’s diagnostic and therapeutic reasoning during a clinical encounter, such as the amount of trust he chooses to place in a radiologist’s report, the cost of a brand medication relative to its generic counterpart, or the extent to which his positive feelings toward a particular patient influence his interpretation of the case. In its current formulation, script theory falls short of explaining how certain contextual and emotional attributes of a case become embedded within developing illness scripts. Recent work, however, has begun to shed light on this area, and may prove helpful for improving the quality of script-based teaching and learning.^42^

Second, it is important to note that, while script theory has been the singular focus of this essay, other useful ‘mental model’ theories have been advanced to explain how information gets stored in and retrieved from health professionals’ minds to influence their reasoning processes during medical encounters. For example, prototype theory posits that diagnostic categories are organized in the mind around a ‘perfect’ abstract case, a prototype that serves as an anchor for other members of the category.^43^ Exemplar theory*,* on the other hand, suggests that categories are developed based on repertoires of previously encountered cases, and that new cases are interpreted based on how similar or not they are to those established reference cases.^7^,^15^ Ultimately, these various mental model theories of knowledge organization – script theory, prototype theory, and exemplar theory, among others – share more commonalities than differences, and an overarching theory of knowledge organization and clinical reasoning will undoubtedly benefit from insights from each.

Finally, using a script-based approach to health professions education presents an unavoidable conundrum. Script activation, and in some cases data interpretation, tends to occur below the threshold of conscious awareness in experienced clinicians. Both clinical teachers and learners may therefore find it difficult to articulate precisely *why* a particular script or scripts leap to mind in a given circumstance. Experienced clinicians may not be aware, for example, of the influence that subtle cues, such as a patient’s facial expression or skin color, have in triggering the mobilization of certain scripts in their minds. It may be even more difficult for them to explain how the setting in which they work affects which scripts they activate and how they interpret them. We submit that this elephant in the room does not render our endeavor to foster script-consciousness pointless; rather, it places bounds on its potential, and serves to remind learners and educators that script building remains an individual exercise influenced by idiosyncratic experience.

**Teaching strategies based on script theory**

**Problem-based learning**: Active elaboration of knowledge through clinical problem-solving leads to better organization of knowledge into illness scripts, facilitating future retrieval

**Self-explanations**: Even without feedback, self-explanations generated during reading or problem-solving lead to deeper learning and enhanced diagnostic reasoning

**Concept mapping**: By depicting how concepts are associated in their minds, learners can render knowledge links visually apparent to their teachers

**Early patient exposure**: Provides learners with opportunities to develop illness scripts for a wide spectrum of clinical presentations from an early stage of training

**Think-aloud strategies**: Can render detailed contents of expert illness scripts transparent for the benefit of learners

**Script-based reading:** Learners can be directed toward relevant sources of information and encouraged to read in a manner that promotes acquisition and linking of conceptual knowledge into illness scripts

**Script-based questioning**: Bedside questions could be framed in such a way as to reinforce associative learning and illness script development

**Direct observation of performance:** Direct observation of clinical evaluations and case presentations in the clinical setting can be a valuable source of evidence of illness script development

**Test-enhanced learning:** Certain assessments, such as script concordance tests and clinical reasoning problems, can be used to improve retention for and organization of study material

## References

[1] Schank RC, Abelson R (1997). Scripts, plans, goals, and understanding.

[2] Frith C (2007). Making up the mind: how the brain creates our mental world.

[3] Monteiro S, Norman GR (2014). Diagnostic reasoning: where we’ve been, where we are going. Teach Learn Med.

[4] Custers EJFM (2014). Thirty years of illness scripts: theoretical origins and practical applications. Med Teach.

[5] Gardner H (1987). The mind’s new science: a study of the cognitive revolution.

[6] Nelson K (1986). Event knowledge: structure and function in development.

[7] Custers EJFM, Regehr G, Norman G (1996). Mental representations of medical diagnostic knowledge: a review. Acad Med.

[8] Feltovich PJ, Barrows HS, Schmidt H, De Volder ML (1984). Issues of generality in medical problem solving. Tutorials in problem-based learning: a new direction in teaching the health professions.

[9] Grant J, Marsden P (1988). Primary knowledge, medical education and consultant expertise. Med Educ.

[10] Charlin B, Tardif J, Boshuizen HPA (2000). Scripts and medical diagnostic knowledge: theory and applications for clinical reasoning instruction and research. Acad Med.

[11] Charlin B, Boshuizen H, Custers E, Feltovitch P (2007). Scripts and clinical reasoning. Med Educ.

[12] Barrows HS, Tamblyn R (1980). Problem-based learning: an approach to medical education.

[13] Croskerry P (2009). A universal model of diagnostic reasoning. Acad Med.

[14] Pelaccia T, Tardif J, Triby E, Charlin B (2011). An analysis of clinical reasoning through a recent and comprehensive approach: the dual process theory. Med Educ Online.

[15] Norman GR (2005). Research in clinical reasoning: past history and current trends. Med Educ.

[16] Dolmans D, De Grave W, Wolfhagen I, van der Vleuten CPM (2005). Problem-based learning: future challenges for educational practice and research. Med Educ.

[17] Kassirer JP (2010). Teaching clinical reasoning: case-based and coached. Acad Med.

[18] Chi MTH, Bassock M, Resnick LB (1989). Learning from examples via self-explanations. Knowing, learning, and instruction: essays in honor of Robert Glaser.

[19] Chi MTH, Glaser R (2000). Self-explaining expository texts: the dual processes of generating inferences and repairing mental models. Advances in instructional psychology.

[20] Chamberland M, St-Onge C, Setrakian J (2011). The influence of medical students’ self-explanations on diagnostic performance. Med Educ.

[21] Novak JD, Gowin DB (1984). Learning how to learn.

[22] Torre D, Durning S, Daley B (2013). Twelve tips for teaching with concept maps in medical education. Med Teach.

[23] Tardif J (1997). Pour un enseignement stratégique - L’apport de la psychologie cognitive.

[24] Schmidt HG, Boshuizen HP (1993). On acquiring expertise in medicine. Educ Psychol Rev.

[25] Schmidt HG, Rikers RMJP (2007). How expertise develops in medicine: knowledge encapulation and illness script formation. Med Educ.

[26] Ahn W, Brewer WF, Mooney RJ (1992). Schema acquisition from a single example. J Exp Psychol Learn Mem Cogn.

[27] Custers EJFM, Boshuizen HPA, Schmidt HG (1998). The role of illness scripts in the development of medical diagnostic expertise: results from an interview study. Cognition Instruct.

[28] Bowen JL (2006). Medical education: educational strategies to promote clinical diagnostic reasoning. N Engl J Med.

[29] Kost A, Chen FM (2015). Socrates was not a pimp: changing the paradigm of questioning in medical education. Acad Med.

[30] Kilminster S, Cottrell D, Grant J, Jolly B (2007). AMEE Guide No. 27: ffective educational and clinical supervision. Med Teach.

[31] Audétat MC, Laurin S (2010). Supervision of clinical reasoning: methods and a tool to support and promote clinical reasoning. Can Fam Physician.

[32] Audétat MC, Laurin S, Sanche G (2013). Clinical reasoning difficulties: a taxonomy for clinical teachers. Med Teach.

[33] Bordage G, Connell KJ, Chang RW, Gecht MR, Sinacore JM (1997). Assessing the semantic content of clinical case presentations: studies of reliability and concurrent validity. Acad Med.

[34] Larsen DP, Butler AC, Roediger HL (2008). Test-enhanced learning in medical education. Med Educ.

[35] Kreiter CD, Bergus G (2009). The validity of performance-based measures of clinical reasoning and alternative approaches. Med Educ.

[36] Charlin B, Brailovsky C, Leduc C, Blouin D (1998). The diagnosis script questionnaire: a new tool to assess a specific dimension of clinical competence. Adv Health Sci Educ.

[37] Lubarsky S, Dory V, Duggan P, Gagnon R, Charlin B (2013). Script concordance testing: from theory to practice: AMEE guide No. 75. Med Teach.

[38] Groves M, Scott I, Alexander H (2002). Assessing clinical reasoning: a method to monitor its development in a PBL curriculum. Med Teach.

[39] West DC, Pomeroy JR, Park JK, Gerstenberger EA, Sandoval J (2000). Critical thinking in graduate medical education: a role for concept mapping assessment?. JAMA.

[40] Woods N (2007). Science is fundamental: the role of biomedical knowledge in clinical reasoning. Med Educ.

[41] Hesketh EA, Bagnall G, Buckley EG (2001). A framework for developing excellence as a clinical educator. Med Educ.

[42] Durning SJ, Artino AR, Schuwirth L, van der Vleuten C (2013). Clarifying assumptions to enhance our understanding and assessment of clinical reasoning. Acad Med.

[43] Bordage G (2007). Prototypes and semantic qualifiers: from past to present. Med Ed.

